# Exploring Chinese middle-class parents’ challenges and coping strategies of dialogic reading in a digital EFL context

**DOI:** 10.3389/fpsyg.2025.1714663

**Published:** 2026-01-05

**Authors:** Lili Zhao, Xiangli Cai, Limin Qin

**Affiliations:** 1 English Department, University of Jinan, Jinan, China; 2 School of Culture and Communication, Shandong University at Weihai, Weihai, China

**Keywords:** Chinese middle-class parents, coping strategies, dialogic reading, digital storybooks, parental challenges

## Abstract

**Introduction:**

Against the backdrop of English learning enthusiasm in China, many Chinese parents are using digital storybooks to support their children’s early English as a Foreign Language (EFL) learning. While previous research has demonstrated the benefits of dialogic reading of digital storybooks, the challenges parents encounter in a non-native English speaking context are under-explored.

**Methods:**

Employing parent–child reading videotaped recordings, reflective journals, and semi-structured interviews, this qualitative study collected data from 18 Chinese parent–child dyads over 8 weeks. Thematic analysis was used to explore parents’ challenges and coping strategies in dialogic reading.

**Results:**

The findings demonstrated that Chinese middle-class parents experienced two major challenges. Parent-related challenges were characterized by the self-limitation of the communicative MKO role, a conflict between high cognitive load and strategy mastery, and time constraints. Child-related challenges stemmed from a mismatch between the child’s ZPD and dialogic strategy demands, as well as attention competition in the digital environment. In response, parents utilized self-scaffolding to equip themselves as competent MKOs. Furthermore, the parents and the digital English storybooks jointly provided dual scaffolding for children’s reading and English language acquisition.

**Discussion:**

By exploring these challenges and coping strategies, this study extends Sociocultural Theory by expanding the concepts of the MKO and Scaffolding in the digital EFL context. It also provides practical insights for parental training, dialogic reading implementation and the design of digital storybook Apps.

## 1 Introduction

In the context of globalization, English proficiency is increasingly viewed as a symbol of cultural and social capital in China (Hu and McKay, 2012). Thus, many parents initiate their children’s English as a Foreign Language (EFL) learning from a very young age (Zeng and Yung, 2025). This phenomenon, often termed “English fever,” is prominent among the burgeoning middle class, whose members invest significant resources to ensure their children gain a competitive edge in a globalized future (Hu and McKay, 2012). Within this trend, parent-led home literacy activities have become a critical component of early language education (Ge, 2023). However, parents’ limited English proficiency often impedes the fluent delivery of English storybook reading, which requires additional support (Liu, 2022).

To address this challenge, digital storybooks have emerged as a potential solution. With enhancement features such as audio narrations, highlighted texts, and pronunciation demonstrations, these digital resources can assist parents with their own language use and provide rich input for children (Cordes et al., 2023; Savva et al., 2022; Yang et al., 2022). However, research indicates that unsupervised use of digital storybooks can have negative effects, such as attention distraction or physical harm (Eggleston et al., 2022; Kucirkova and Flewitt, 2022). Therefore, in order to harness the benefits of digital reading while mitigating its risks, effective parental mediation is essential, such as parent–child reading.

Parent–child dialogic reading, an evidence-based interactive reading approach, is likely to be a powerful strategy to enhance such parental guidance (Whitehurst et al., 1988). Dialogic reading is used to keep children engaged with story content through interactive conversation, making the combination of dialogic reading with digital storybooks appear to be an ideal approach to promote children’s English language ability (Cordes et al., 2023; Strouse et al., 2023).

However, this combination may pose challenges for non-native English-speaking parents. Previous research confirms that parents in the EFL context not only face cultural and language challenges (Zeng and Yung, 2025) but also struggle with implementing dialogic reading strategies (Zevenbergen et al., 2018). However, few studies have explored the challenges when dialogic reading interacts with digital storybooks in the EFL context. The gap is significant because these challenges may affect parents’ dialogic reading strategy use and subsequent language outcomes (Liu, 2022). Therefore, to leverage the benefits of digital dialogic reading, it is essential to first understand the comprehensive challenges parents encounter and coping strategies they develop. To address this gap, this study explores the following research questions:

1. What challenges did Chinese parents encounter when implementing dialogic reading strategies with their young children in a digital EFL context?
2. What strategies did Chinese parents employ to cope with these challenges during the implementation process?

## 2 Literature review

Dialogic reading, first introduced by Whitehurst et al. (1988), represents an interactive approach where adults engage children in meaningful conversations about stories through specific questioning and feedback techniques. The techniques in dialogic reading with children include the PEER sequence and CROWD prompts (Zevenbergen and Whitehurst, 2003). The PEER strategy follows the sequence: (1) Prompt: encourage the child to say something about the reading materials; (2) Evaluate: give feedback about the child’s answer; (3) Expand: add information to the child’s response or use other ways to supplement children’s answer; (4) Repeat: ask the child to repeat to make sure the expanded or correct response can be memorized. The CROWD prompts include: (1) Completion prompts: leave a blank for children to fill in at the end of a sentence, especially used in books with repetitive patterns; (2) Recall prompts: ask the child to recall what has been read in a book for the facilitation of their memorization about the story event; (3) Open-ended prompts: encourage the child to talk about the story his own words based on illustrations of the book; (4) Wh-prompts: use Wh-questions (what, where, when, why and how questions) to elicit more information about the book; (5) Distancing prompts: associate the book with their real life and experience.

### 2.1 The efficacy of dialogic reading of digital storybooks

Studies on dialogic reading of digital storybooks have yielded fruitful results on children’s language outcomes in the first language context (Korat and Shneor, 2019; Strouse et al., 2023; Troseth et al., 2020). For example, Korat and Shneor (2019) conducted a study with a sample of 128 preschoolers from 11 Hebrew-speaking kindergartens in Israel. The results suggested that reading an e-book accompanied by a dictionary and maternal mediation was optimal for receptive and expressive vocabulary learning, phonological awareness, and word reading. In addition, Strouse et al. (2023) reported that the researcher-designed built-in prompts in the digital storybooks can boost parent–child talk during their reading. Furthermore, Troseth et al. (2020) investigated parent–child talk and their engagement after implementing an enhanced e-book reading intervention among 32 parents and their children in the US. The enhanced e-book was embedded with an interactive character, Ramone, who modelled dialogic reading strategies for parents. The result indicated that parents verbalised significantly more and evoked significantly more story-related reading activities than parents in control groups.

Given its success in first language settings, researchers have increasingly explored the potential of dialogic reading for second/foreign language learners. Studies in classroom settings have consistently shown that dialogic reading can be an effective tool for teachers to increase young learners’ vocabulary acquisition and oral fluency in a second language (Chow et al., 2023). Extending this line of inquiry to the home environment, the study by Yang et al. (2022) investigated the effects of bilingual prompts (both English and Chinese) embedded in e-book Apps when used by Chinese parents reading to children aged three to seven. Their findings indicated that this approach also facilitated children’s story comprehension and yielded significant gains in English language outcomes.

### 2.2 Digital storybook: affordance and constraint

The proliferation of digital technologies has introduced a new dimension to family literacy. Digital books and reading applications offer a range of “affordances” that can potentially support EFL learning. Features such as embedded dictionaries, audio narration by native speakers, and text highlighting can provide immediate linguistic support, potentially compensating for parents’ own language limitations (Reich et al., 2016). Interactive elements, such as animations and games related to the story, can also enhance engagement and motivation for young learners (Eutsler and Trotter, 2020). For EFL families, these features could serve as a valuable scaffold, providing accurate language models and enriching the reading experience.

Conversely, the enhanced features of digital storybooks can also pose challenges. Research indicated that interactive elements can distract children and parents from the story’s content, leading to shallower processing of the text (Kucirkova and Flewitt, 2022). Moreover, the nature of parent–child interaction can shift when using digital devices (Munzer et al., 2019; Strouse and Ganea, 2017). For instance, Munzer et al. (2019) found that when reading e-books, parent-toddler talk was more focused on behavior management than on story content. This phenomenon, sometimes termed “technoference,” can reduce the frequency and quality of the rich, content-focused dialogue (Strouse and Ganea, 2017).

### 2.3 Early EFL education and parental involvement in China

Globally, children are beginning to learn English as a foreign language (EFL) at a younger age (Prošić-Santovac and Radović, 2018). Parents were expected to take responsibility to invest in their children’s learning (Carmel, 2022). This trend is especially prominent in China, which embraced neoliberalism to enhance its global economic competitiveness. Neoliberalism was characterized by the belief in free markets (Harvey, 2005). This neoliberal discourse, amplified by public media, has greatly raised the status of English in Chinese society (Zeng and Yung, 2025). Consequently, English proficiency has been viewed as essential for academic achievement and upward mobility (Hu and McKay, 2012). These parents, especially middle-class parents who are driven by a desire to secure a competitive advantage for their children, invest heavily in early EFL education (Carmel, 2022). Despite their high aspirations, non-native English-speaking parents in China encountered challenges in facilitating their children’s EFL learning, particularly linguistic limitations (Liu, 2022; Zeng and Yung, 2025).

Overall, while dialogic reading is an evidence-based intervention, its research is predominantly situated in native-speaking contexts (Strouse et al., 2023; Troseth et al., 2020). Meanwhile, while digital storybooks offered valuable scaffolds for parents (Reich et al., 2016), they also introduced the risk of reducing dialogue quality (Strouse and Ganea, 2017). Furthermore, EFL middle-class parents are under pressure to transmit English as cultural capital (Hu and McKay, 2012) and encounter their own linguistic challenges (Liu, 2022). However, few studies have explored the challenges from the multiple aspects of dialogic reading of digital English storybooks in EFL contexts. It is also unknown how parents coped with challenges. In order to address this gap, this study will explore the challenges and coping strategies in the dialogic reading of digital English storybooks.

### 2.4 Theoretical framework

This study is grounded in Sociocultural Theory (Vygotsky, 1978), which posits that learning is a fundamentally social process mediated by and with more knowledgeable others and cultural tools. The More Knowledgeable Other (MKO) is the person who has a higher level of knowledge, skills, or experience. In contrast, Significant Others are defined by emotional closeness or social importance rather than expertise. In this study, parents assumed the MKO role not because of their emotional relationship with the child, but because they were expected to guide the cognitive and linguistic aspects of digital dialogic reading. The cultural tools are the symbolic and physical artifacts of a society, such as language, writing, or technology, that mediate thought and social activity. Central to Sociocultural Theory is the concept of the Zone of Proximal Development (ZPD), which describes the crucial space between what a learner can achieve independently and what they can accomplish with guidance. The primary role of the MKO is to provide targeted support within this ZPD. This process is best conceptualized as Scaffolding. This term was coined by Wood et al. (1976) to describe the temporary, adjustable assistance an expert provides to help a novice complete a task they could not manage alone.

In the dialogic reading of digital English storybooks, parents are regarded as MKOs and expected to provide scaffolding within their children’s ZPD. In this study, the digital storybooks also function as technological scaffolds. With features like audio, visual, and translation, they are designed to help the child bridge the gap within their ZPD. However, a central tension arises as these non-native speaking parents may encounter their own linguistic challenges (Liu, 2022; Zeng and Yung, 2025), which can affect their ability to effectively perform the MKO role. This raises the question of how the scaffolding of the digital storybook is utilized in this complex context. Therefore, this study employs Sociocultural Theory as a lens to not only investigate how parents enact their MKO role, but also to analyze how digital storybooks provide scaffolding for parent–child dialogic reading.

## 3 Research method

### 3.1 Participants

Participants in this study consisted of 18 Chinese parent–child dyads recruited through purposive and convenience sampling strategies (see Table 1). Recruitment was conducted via WeChat groups, kindergarten principals’ networks, and QQ groups in the capital city of an eastern province in mainland China. Participants were selected based on four criteria: (i) children aged 5–6 years, as this age group can provide meaningful responses to higher-level questions due to their increased vocabulary (Lonigan et al., 2007); (ii) children with no reported learning, cognitive, or physical disabilities; (iii) families with access to digital devices (touch tablets, smartphones, or computers); and (iv) both parents and children having Chinese as their only home language. After expressing interest, parents were invited to a WeChat group to learn about the program duration, procedure, and potential challenges. Final participant selection was based on parents’ willingness and compliance with the research criteria. This sample was deemed sufficient to provide the “thick description” (Lincoln and Guba, 1985) required to adequately address the research questions.

**Table 1:** The demographic profile.

Variable	Category	*N*	Percentage
Parent’s gender	Mother	16	88.9%
	Father	2	11.1%
Parents’ age	34–43 years old		
Child’s gender	Boy	14	77.8%
	Girl	4	22.2%
Child’s age	5–6 years old		
Parents’ English level	CET-4	11	61.1%
	CET-6	5	27.8%
	TEM-8	2	11.1%
Parents’ educational level	Bachelor	13	72.2%
	Master	3	16.7%
	PhD	2	11.1%
Household income	RMB 100,000–300,000		

The child participants consisted of 14 boys and four girls, ranging in age from 5 to 6 years. They were mostly the second child in the family (*N* = 15). This is related to China’s shift from the long-standing one-child policy to the universal two-child policy in 2016, which led to a noticeable rise in second-born children (Li et al., 2019). Most of the parent participants were mothers (*N* = 16) with ages ranging from 34 to 43 years old. All of them have a bachelor’s degree, with three holding master’s degrees and two possessing doctoral degrees. Their English proficiency was proven by passing the most influential English exams held in China, with two parents passing the Test for English Majors, band eight (TEM-8), five parents passing the College English Test, band six (CET-6), and 11 parents passing the College English Test, band four (CET-4). All families had an annual household income of RMB 100,000–300,000. According to national statistical benchmarks, this range aligns with the urban middle-class income group in China, who typically possess stable educational and socioeconomic resources (National Bureau of Statistics of China, 2023).

These urban middle-class families represent an information-rich sample due to their distinctive sociocultural positioning within China’s early EFL landscape. Prior research shows that Chinese middle-class parents typically possess higher educational attainment and strong aspirations for early English learning, which drive them to invest heavily in children’s language development (Carmel, 2022; Hu and McKay, 2012; Zeng and Yung, 2025). Their socioeconomic stability affords them access to digital devices, paid Apps, and extracurricular resources, conditions known to enhance opportunities for home-based English learning (Reich et al., 2016; Korat et al., 2022). At the same time, studies indicate that even educated Chinese parents often experience partial English proficiency, leading to anxiety and uncertainty during shared reading (Liu, 2022; Zou, 2020). Combined with the intensified work-family pressures experienced by Chinese middle-class mothers in particular (Lin et al., 2020), these factors create unique cognitive and emotional tensions that shape how dialogic reading is enacted. Therefore, this group offers rich, practice-based insights into both the affordances and constraints of digital dialogic reading in contemporary Chinese EFL households.

### 3.2 Reading materials

Eight digital storybooks were selected from Audio English Storybook App, a Chinese digital App equipped with over 1,000 popular English picture books for children over 4-years-old. It is embedded with audio narration, visual picture, text highlighting, and a translation function. Books from *the Oxford Reading Tree series (levels 1–3), I Can Read Series* were chosen based on Vygotsky’s (1978) ZPD theory (see Table 2). This selection aimed to match children’s English proficiency level, enabling them to engage with the content through parental support. As the most popular picture book series for home reading in China, these books, featuring simple, repetitive words and amusing story plots, could facilitate parent–child dialogue while maintaining children’s interest (Yadi, 2020).

**Table 2:** Reading materials for the 8-week intervention.

Intervention week	Book name
Week 1	New Trees: Oxford Reading Tree (Brychta, 2003b)
Week 2	Kipper’s Birthday: Oxford Reading Tree (Brychta, 2003a)
Week 3	The Wobbly Tooth: Oxford Reading Tree (Brychta, 2005c)
Week 4	The Big Egg: Oxford Reading Tree (Brychta, 2005a)
Week 5	The New Gingerbread Man: Oxford Reading Tree (Brychta, 2005b)
Week 6	The Snowman: Oxford Reading Tree (Brychta, 2003c)
Week 7	Biscuit Goes to School: I Can Read Series (Capucilli, 2003a)
Week 8	Biscuit’s Big Friend: I Can Read Series (Capucilli, 2003b)

### 3.3 Data collection

Drawn on a qualitative methodology, this study collected data via parent–child reading videotaped recordings, parents’ reflective journals, and semi-structured interviews. First, to capture authentic parent–child dialogic reading interactions, video recordings were collected from each participating dyad over an eight-week period. Each parent–child dyad was requested to record one dialogic reading session per week, with each session lasting approximately 10 min. Participants were instructed to conduct the reading sessions in their natural home environment, such as the living room or bedroom, and at their usual reading time, before bedtime or after dinner. These recordings were later transcribed verbatim to create a detailed record of the dialogue to explore parents’ challenges and coping strategies.

In addition, parents’ reflective journals were employed to gather data about the challenges parents encountered and coping strategies. The reflective journal protocol included the following items: (1) the book name; (2) the reading date; (3) the challenges parents encountered; (4) the strategies parents used to cope with the challenges. Parents were instructed to complete one reflective journal per week throughout the eight-week implementation period. To ensure consistency of the details, parents were provided with a template for guidance (see Appendix B).

Furthermore, semi-structured interviews were conducted to explore Chinese parents’ challenges and coping strategies upon completion of the dialogic reading program. To ensure the interview questions were appropriate, the protocol was reviewed by two senior researchers in child language acquisition and family education. This review helped confirm that the questions were clear and open-ended. The interview protocol included (1) overall experience of dialogic reading of digital storybooks; (2) challenges parents encountered during digital dialogic reading; (3) parents’ strategies to overcome challenges (see Appendix A). The interviews were conducted in Chinese online to allow participants to express their experiences fully. Each interview lasted approximately 30–45 min and was audio-recorded for subsequent analysis. During and after each interview, the researchers also wrote field notes to capture observations about the participants’ non-verbal messages that provided additional context to their verbal responses. The interviews were transcribed verbatim and then member checking was conducted by sharing interview transcripts with participants for verification.

### 3.4 Procedure

The parent–child dialogic reading program in this study involved 18 Chinese families reading at home. After obtaining signed consent forms from parent participants, a dialogic reading strategy training session was implemented for all the parents. Initially, a 15-min video on dialogic training was used to coach parents. Translation in Chinese and additional explanations were provided to assist parents’ comprehension. Next, the researcher demonstrated the dialogic reading techniques of one digital English storybook by sliding in both English and Chinese. The instructions of techniques were displayed page after page so that parents could easily follow. After watching the video and slide demonstration, parents practiced the strategies and obtained feedback from the researcher to ensure they could master these reading skills. Subsequently, parents were asked to download the Audio English Storybook App to access the targeted books in this study, and then were required to read the storybook using the demonstrated dialogic reading strategies. Parents should read these eight books with their children each week at a convenient time and location. One parent–child reading session needed to be video recorded weekly by smartphones or other recorders and submitted. Moreover, parents were requested to write the reflective journals weekly and send them to the researcher. To support implementation fidelity during the 8-week period, the researcher also provided parents with ongoing feedback on their strategy use after reviewing their weekly video recordings. Upon completion of the eight-week implementation, an online semi-structured interview was conducted with 18 parent participants in Chinese and lasted approximately 30–45 min to explore parents’ challenges and coping strategies during the dialogic reading program.

### 3.5 Data analysis

All Chinese data were first transcribed verbatim and translated into English by two bilingual researchers to ensure accuracy. Thematic analysis was employed to analyze the video recording transcripts of parent–child dialogic reading, semi-structured interviews, and reflective journals based on Braun and Clarke’s (2006) approach. Using MAXQDA 22, the analysis followed six steps: (1) Data immersion through repeated reading to understand parents’ experiences with digital dialogic reading; (2) Initial coding. We combined inductive and deductive approaches to create codes (see Miles et al., 2014). A primarily inductive, bottom-up process was used to ensure codes were grounded in the data. This allowed descriptive codes, such as “parents’ limited vocabulary” and “child’s lack of attention,” to emerge directly from the participants’ accounts. Meanwhile, a deductive approach, informed by our theoretical framework, was used to interpret and categorize the coping strategies that parents reported; (3) Theme development by grouping codes into coherent patterns representing major challenge categories and adaptive responses; (4) Theme refinement through constant comparison to ensure themes accurately reflected parents’ challenges and coping strategies in EFL dialogic reading contexts; (5) Theme definition to capture the essence of challenges and coping strategies; (6) Final interpretation connecting findings to the broader context of dialogic reading and home literacy in Chinese families.

### 3.6 The quality of research

Ethical approval was obtained from the first researcher’s institutional review board prior to the study. All participants were informed of the study’s purpose, procedures, and their right to voluntary participation and withdrawal. Following this, all parents provided signed written informed consent. To maintain participant confidentiality, parent participants were labeled as P1, P2, P18, while child participants were labeled as C1, C2, C18. Furthermore, all digital files were stored on a password-protected hard drive and only accessed by the researchers.

To establish the trustworthiness of the findings (Lincoln and Guba, 1985), several strategies were employed. First, data triangulation was achieved by collecting and cross-verifying data from three distinct sources: parent–child reading videotaped recordings, parents’ reflective journals, and semi-structured interviews. This allowed for a comprehensive and robust understanding of the participants’ experiences. Second, member checking was performed. Participants were invited to confirm the accuracy of their interview transcripts. Finally, transparency was ensured through the use of “thick description,” providing rich, raw data excerpts in the results to support and substantiate the identified themes.

In addition, the fidelity of the implementation was monitored. The researchers reviewed the weekly video recordings. These videos provided direct evidence of parent–child interaction and allowed researchers to confirm that parents were implementing dialogic reading strategies as trained. Meanwhile, the reflective journals required parents to document their experiences to confirm their active engagement. Furthermore, based on these videotaped recordings and reflective journals, the researchers provided personalized feedback to each parent and offered suggestions to help them refine their dialogic reading strategies. This ongoing monitoring and feedback ensured parents remained actively engaged in the dialogic reading process throughout the study.

Finally, the researchers maintained a reflexive stance regarding positionality. As parents and experienced dialogic reading guides, the researchers possessed an “insider” understanding. This facilitated rapport and a nuanced comprehension of the participants’ challenges. To mitigate the potential bias inherent in this role, a reflexive stance was maintained throughout the research process, and the “outsider” perspective from peer debriefing was used to examine the findings.

## 4 Results

Through an analysis of videotaped recording transcripts, reflective journals, and interviews, this study revealed challenges that Chinese parents encountered and coping strategies in the dialogic reading of digital English storybooks.

### 4.1 Challenges

In this study, Chinese parents mainly encountered two major challenges of dialogic reading of digital English storybooks: parent-related challenges and child-related challenges.

#### 4.1.1 Parent-related challenges

The analysis revealed that parent-related challenges were categorized into three major themes: the self-limitation of the communicative MKO role, a conflict between high cognitive load and strategy mastery, and time constraints under social pressure (see Table 3).

**Table 3:** Parent-related challenges.

Major challenges	Manifestation and impact	Code examples	Frequency
Self-limitation of the communicative MKO role	Linguistic limitations caused them to self-limit their interaction, impairing their role as the MKO.	“I can only read the words from the storybooks, but words beyond the books are difficult” (P5)	*N* = 16
Conflict between high cognitive load and strategy mastery	High cognitive load leads to difficulty in mastering multiple strategies and applying them naturally.	“I find it overwhelming to use all the different types of questions and prompts during a reading session” (P11)	*N* = 15
Time constraint under social pressure	Parents bear multiple responsibilities, leading to reading time being squeezed.	“It’s really challenging when both kids need attention” (P5)	*N* = 10

Firstly, the frequently reported challenge was the parents’ linguistic production anxiety and lack of confidence, which led to the self-limitation of their communicative MKO role (*N* = 16). This limitation included vocabulary or grammar constraints, which led to a lack of confidence and anxiety. As P5 stated, “I can only read the words from the storybooks, but words beyond the books are difficult” (P5, Interview). This uncertainty extended to their worries about pronunciation, as P8 confessed, “I just ask my child to repeat the words in the App, but I’m not sure if his pronunciation is correct” (P8, Reflective Journal). Notably, this anxiety was not confined to those with lower English proficiency. Even P3, who had passed the CET-6 university English exam, felt similarly constrained, “When I want to ask deeper questions or encourage more discussion, I sometimes hesitate because I’m worried about making grammatical mistakes” (P3, Reflective Journal).

The second major challenge stemmed from the high cognitive load and conflict with adult learning principles, resulting in significant difficulty in mastering and translating dialogic reading strategies into natural practice (*N* = 15). Twelve parents reported feeling overwhelmed by having to master and differentiate multiple strategies at once. P9 stated, “It’s too difficult for me to master so many strategies at one time” (P9, Reflective Journal). This theoretical confusion led directly to role confusion in practice. P11 expressed a common struggle, “Even though I can remember the strategies, I find it overwhelming to use all the different types of questions and prompts during a reading session” (P11, Interview).

Specifically, the practical implementation of individual dialogic reading strategies was severely challenged by linguistic barriers and child responsiveness. This was true when attempting the Expand strategy. As P7 explained, “I can do this naturally when reading Chinese books, but when it’s in English, I just cannot find the right words” (P7, Reflective Journal). Another example is P15, who minimized the use of Completion prompts because her child “had not encountered this type of English interaction before and often failed to respond” (P15, Interview). Similarly, P8 acknowledged this barrier with Open-ended prompts, stating, “The only one I used less was open-ended prompts, because I did not know how to ask more” (P8, Interview). In addition, P4 reflected on her limited range of evaluative language for the Evaluate strategy: “I found that I used the Evaluate strategy more and more often, but my evaluation was a bit monotonous” (P4, Reflective Journal).

Thirdly, time constraint under social competitive pressure emerged as a challenge. Participants reported they bore multiple responsibilities, including house chores, children’s assignments and workload, particularly for families with multiple children. These demands often resulted in inconsistent implementation or complete omission of scheduled reading activities. As P5 described: “It’s really challenging when both kids need attention. While I’m helping my elder one with math homework, the younger one is asking for story time” (P5, Reflective Journal). Parents also reported time constraints in implementing dialogic reading strategies. As P7 noted, “At the initial stage, time was very limited because I was not skilled at using the digital storybook App. I had to check the strategy sheet, use more English, and operate the app all at once. So, I could not manage everything at the beginning” (P7, Interview).

#### 4.1.2 Child-related challenges

The major child-related challenges encompassed a mismatch between the child’s ZPD and dialogic reading strategy demands, as well as attention competition in the digital environment (see Table 4).

**Table 4:** Child-related challenges.

Challenges	Sub-theme	Code examples	Frequency
Child-related challenges	Mismatch between child’s ZPD and dialogic reading strategy demands	“Since pages had longer, faster sentences, she struggled to understand when reading or listening alone, leading to reduced interest.”	*N* = 18
	Attention competition in the digital environment	“My son just wants to explore every function, constantly clicking buttons to see what happens, and does not pay attention to the story itself.”	*N* = 12

Firstly, the implementation process was hindered by a mismatch between the child’s ZPD and dialogic strategy demands, which primarily manifested as children’s linguistic limitations. For example, as P10 explained: “Since pages had longer, faster sentences, she struggled to understand when reading or listening alone, leading to reduced interest” (P10, Reflective Journal). This can be supported by a video transcript. When P9 used an open-ended prompt like, “Why do you like winter?,” her child could only respond with the single word “snowman” and was unable to construct a full sentence. This made it difficult to implement dialogic reading strategies that require longer, more expressive responses. P1 expressed, “She still needed me to translate much of the content, and sometimes forgot words she had learned” (P1, Interview).

Secondly, children showed distractibility and engaged in off-task behaviors. Even when initially cooperative, focus could wander. As P6 noted, “But once she accepted it, I found that she did not seem as focused and would get distracted. So she would want to do other things” (P6, Interview). The sources of distraction varied. Sometimes it was the digital environment itself, such as the impulse to swipe the screen: “At the beginning, when I finished reading a page, the child would swipe with his finger. I had to hold his hand to stop him” (P7, Interview). P14 also complained, “My son just wants to explore every function, constantly clicking buttons to see what happens, and does not pay attention to the story itself” (P14, Interview). Furthermore, the story content sparked internal distractions, “When I read the story about a dog with my child, he was preoccupied and constantly expressed his desire to have a pet dog (P11, Reflective Journal). Finally, high cognitive load could also lead to distraction, as P6 explained that difficulty understanding longer sentences resulted in reduced interest (P6, Reflective Journal).

### 4.2 Coping strategies

Faced with these challenges, different scaffolding was provided: self-scaffolding, where parents first supported their own competence and confidence as learners, and dual scaffolding, where parents and digital English storybooks provided support for parent–child dialogic reading (see Table 5).

**Table 5:** Coping strategies.

Coping strategies	Sub-theme	Code example	Frequency
Self-Scaffolding	Develop dialogic reading skills	“I practiced certain strategies for the first time, and then another strategy, and gradually I mastered these strategies.”	*N* = 14
	Peer support	“I watched other parents’ demonstration videos in the WeChat group and asked why they did it that way, which helped me learn quickly.”	*N* = 4
	Develop Technological and Linguistic skills.	“I click all the buttons to see what happens, then I will know how to operate them.” “I searched for Natalie’s storybook accompaniment materials, recorded a few especially inspiring questions, and then set more meaningful questions for the child.”	*N* = 3
Dual Scaffolding	Parents’ scaffolding	Bilingual Support: “I translate the words and sentences page by page and then ask her to describe the pictures.”	*N* = 16
		Contextual Management Strategies: “making reading a compulsory thing before bedtime not only protected the time for reading but also transformed it into an anticipated and comforting family ritual.”	*N* = 10
	Digital English Storybook’s Scaffolding	“What did Dad see? Look at the picture.” “What is the puppy in English? Listen again”	*N* = 18

#### 4.2.1 Parents’ self-scaffolding: the parent as a learner

A notable finding is that parents had to scaffold themselves first. This involved developing dialogic reading skills, peer support, and developing technological and linguistic skills.

Firstly, parents developed skills to cope with the implementation difficulties of the dialogic reading strategy. Parents first engaged in self-directed learning. For example, four parents created physical aids for strategy implementation, with P11 noting: “I made a list of all the strategies and typed them into a piece of paper and reviewed them. I refer to them when reading with my child” (P11, Reflective Journal). In addition, three parents also adopted a progressive learning approach, as explained by P15, “I practiced certain strategies for the first time, and then another strategy, gradually I mastered these strategies” (P15, Reflective Journal).

Secondly, the parent peer support played a role through video demonstrations and WeChat groups. As P13 shared, “I watched other parents’ demonstration videos in the WeChat group and asked why they did it that way, which helped me learn quickly” (P13, Interview). This support also provided motivation, as P18 reflected, “Sometimes I want to quit, but when I see other parents so enthusiastic about their child’s reading and their children make progress in English proficiency, I would be motivated to continue” (P18, Reflective Journal).

Thirdly, parents also scaffolded their own technological and linguistic skills. They learned to familiarize themselves with the Apps beforehand to manage the technology challenges. As P14 stated, “I click all the buttons to see what happens, then I will know how to operate them” (P14, Interview). It should be noted that two parents (P6; P18) even used AI chatbots as P6 expressed, “Kimi or Doubao to help me construct sentences and boost my confidence in language production” (P6, Interview). Furthermore, parents relied on online resources to design dialogic reading questions and translation tools to support English understanding. As P12 explained, “I searched for *Natalie’s storybook* accompaniment materials, recorded a few especially inspiring questions, and then set more meaningful questions for the child” (P12, Reflective Journal). P18 also explained, “There are some useful reference materials online that offer questions and prompts for specific books we are reading, so I can learn these materials beforehand and incorporate them when reading with my child” (P18, Interview).

#### 4.2.2 Dual scaffolding: supporting dialogic Reading

Parent–child dialogic reading of a digital English storybook was scaffolded by two main ways: parents’ scaffolding and digital English storybook scaffolding.

##### 4.2.2.1 Parents’ scaffolding

Firstly, parents employed their native language (Chinese) to support their dialogic reading of digital English storybooks. All the parents also employed their native language as a scaffold through code-switching and translation. They used Chinese as a bridge language to provide bilingual support, with Chinese-dominant interaction as one approach, where Chinese was primarily used for story discussion while introducing targeted new words in English, as illustrated in P10’s reading (see Excerpt 1):

Excerpt 1: a parent–child dialogic reading video with the book *New Trees* from *Oxford Reading Tree.*

-P10: [Audio plays: Dad gave a tree] 爸爸要种一棵树, 那爸爸现在在干什么? (Dad is going to plant a tree. What is he doing now?) [Wh-prompt].
-C10: 在挖土。(He is digging the soil).
-P10: 用什么挖?(What is he using to dig?) [Wh-prompt].
-C10: 用铲子。(Using a shovel.)
-P10: 对。你看周围还有谁在看啊?(Correct. Who else is watching nearby?) [Wh-prompt].
-C10: 还有小朋友。(There are children.)
-P10: [Audio plays: put it by the shed] 他把树种在一个栅栏旁边，栅栏是shed. (He planted the tree by the shed. 栅栏 means a shed.)
-C10: Shed.

In this excerpt, P10 conducted almost the entire dialogic reading session in Chinese while introducing only the English word “shed.” By employing Chinese to implement the dialogic reading strategies, P10 noted, “I could maintain the meaningful interaction by using Chinese, which can help me to overcome the language barrier.” (P10, Interview).

Furthermore, parents frequently utilized direct English-to-Chinese translation to explain new words or sentences. P15 detailed how she combined these two strategies: “I translate the words and sentences page by page and then ask her to describe the pictures” (P15, Interview). Meanwhile, she also flexibly used code-switching during the interaction (see Excerpt 2).

Excerpt 2: a parent–child dialogic reading video with the book *The Snowman* from *Oxford Reading Tree.*

-P15: She made a snowball, 而且是一个Giant snowball (and it was a giant snowball).
-C15: 什么叫Giant snowball? (What does “Giant snowball” mean?)
-P15: (Stretched out the arms) Giant snowball 就是这么大的 snowball, giant 是巨大的意思。 (Giant snowball means a snowball this big; “giant” means very large.)
-C15: 我去年也做了两个snowballs, 一个大的，一个小的。 (I also made two snowballs last year, one big and one small).
-P15: 是的，你做了 two snowballs, 一个 small snowball, 一个 big snowball, 但是不算 giant snowball. (You made two snowballs, one small snowball, one big snowball, not a giant snowball.)

In this excerpt, P15 clarified the meaning of the targeted word “giant” by direct translation while seamlessly using code-switching to integrate English new words into Chinese sentences. By alternating between English and Chinese, P15 could “overcome my English sentence production obstacle and also increase my child’s English vocabulary exposure” (P15, Interview).

Secondly, parents employed a range of contextual management strategies to create a focused and positive reading environment. Specifically, they also established clear rules with their children regarding screen control. To actively manage their child’s engagement, parents employed creative interactive techniques. For example, P12 used physical interactions to guide her child’s reading to differentiate words “sad” and “happy” (see Excerpt 3).

Excerpt 3: from a parent–child dialogic reading video with the book *The Wobbly Tooth* from *Oxford Reading Tree*.

P12: 妈妈说glad, 你就做 glad的脸型, 我说sad, 你就做 sad 的脸型。我说 upset, 不安, 你就做不安的脸型好不好?那现在来了啊, Sad. (When I say “glad,” you make a glad face. When I say “sad,” you make a sad face. When I say “upset,” you make an upset face, okay? Now, let us start. Sad.)
C12: (made a face of “sad”) 这是sad. This is sad.
P12:下一个glad. (Next, glad.)
C12: Made a face of “glad.”
P12: 好, 下一个upset. Upset不安。 (Ok, next, upset. Upset means uneasy.
C12: Made a face of “upset.”

Moreover, they used games to guide their interaction. For example, P6 invited her son to act as a “little detective”: “咱们俩当一次小侦探，分析分析这个蛋到底是谁的(Let us be little detectives and analyze whose egg this is).” They also leveraged dialogic reading itself for emotional connection, using distancing questions and specific praise like, “You can do it by yourself, that is amazing, please give me five!” (P12, Reflective Journal) to reinforce motivation. Meanwhile, they integrated reading into a predictable daily routine. As P6 explained, “making reading a compulsory thing before bedtime not only protected the time for reading but also transformed it into an anticipated and comforting family ritual” (P6, Interview).

##### 4.2.2.2 Digital English storybooks scaffolding

The digital English storybooks in this study provided technological scaffolding for parent–child dialogic reading. Firstly, all parents relied on the audio playback feature to model correct pronunciation, which compensated for their own linguistic insecurities. For example, when her child pointed to a word, P5 did not pronounce it herself but instructed, “小狗是什么? 再听听 (What is the puppy in English? Listen again)” (P5, Videotaped Recording).

Secondly, visual illustrations were frequently used as a non-linguistic bridge to comprehension. Parents prompted their children to use visual cues to decode sentences, such as P9 asking, “What did Dad see? Look at the picture?” (P9, Videotaped Recording). They also used the cover-page illustrations to initiate low-pressure conversations, such as “Who is on this cover? What color is this bus?” (P4, Videotaped Recording).

Finally, the translation feature of the digital English storybook was employed to overcome specific vocabulary hurdles. This feature served as an essential fallback, ensuring that comprehension barriers did not completely disrupt the flow of the dialogic reading session. P11 described how the translation features became a fallback when a sentence was too difficult:

Because sometimes I wasn’t sure about the meaning of a word, or a sentence was difficult to translate. I tried to look up words, but a single word alone did not connect well with the sentence, so I worried about misleading her. Later, I used the translation features in the App. (P11, Interview)

## 5 Discussion

This study aimed to explore the challenges and coping strategies of parents in implementing the dialogic reading of digital English storybooks. While Sociocultural Theory posits that parents act as MKOs to provide scaffolding within the child’s ZPD, our findings complicate and extend this model in the specific digital EFL context.

### 5.1 Challenges of parents as MKOs

One major finding of this study is that parents were faced with many challenges in the dialogic reading of digital English storybooks in the EFL context, which has challenged their MKO roles.

#### 5.1.1 Parent-related challenges

The findings revealed that these challenges were shaped by mechanisms specific to the Chinese EFL context. Parents’ widely reported linguistic limitations reflected the legacy of China’s exam-oriented English education, which emphasizes grammatical accuracy and written tests over communicative competence (Zou, 2020). As a result, even parents with relatively high English proficiency reported anxiety about pronunciation, grammar, and the adequacy of their explanations. This aligns with previous research showing that non-native English-speaking parents in China and other EFL contexts often feel underprepared to support their children’s English learning at home (Liu, 2022; Seo, 2021; Zeng and Yung, 2025). In this study, such anxiety directly undermined parents’ confidence in assuming the MKO role (Vygotsky, 1978), leading them to avoid more complex prompts or spontaneous elaboration even when they understood the story content.

Difficulties in implementing dialogic reading strategies extended far beyond remembering PEER or CROWD strategies. Parents were required to manage three demanding tasks simultaneously: producing English input, selecting and sequencing dialogic prompts, and operating the digital App in real time. This multiple cognitive load is consistent with Sweller’s (1988) cognitive load perspective and helps explain why parents relied on a limited set of familiar prompts while using higher-level strategies less frequently, such as the Distancing prompt or the Expand strategy. Specifically, the scarcity of the Expand strategy contrasts with its frequent use in L1 contexts (Chang et al., 2023), reflecting how linguistic barriers in the EFL setting inhibit parents from sustaining higher-level questioning. Previous studies have shown that even L1 parents tend to avoid open-ended prompts because of their cognitive demands (Zevenbergen et al., 2018; Zucker et al., 2009). The present finding extends this literature by illustrating how these cognitive demands are intensified when dialogic reading takes place in a digital EFL environment.

Time constraints further compounded these challenges. Following the universal two-child policy, many families faced increased caregiving responsibilities (Hong et al., 2022). At the same time, rapid social change and heightened academic competition have intensified parents’ concerns about educational outcomes (Ng and Wei, 2020; Roskam et al., 2017). Middle-class parents, in particular, experience strong neoliberal pressure to invest heavily in early English learning as a form of cultural capital (Carmel, 2022; Hu and McKay, 2012). Within this sociocultural context, mothers continued to bear the majority of childcare and domestic duties (Li, 2020; Lin et al., 2020), so they struggled to find both the time and mental energy required for sustained, high-quality dialogic reading.

#### 5.1.2 Child-related challenges

The finding revealed that children’s challenges in dialogic reading of digital English storybooks were shaped by developmental, cognitive, and technological factors specific to Chinese digital EFL homes. Previous studies have documented that young EFL learners often struggled with minimal English exposure (Liu, 2022; Seo, 2021), but our findings showed that children’s difficulties extended beyond vocabulary gaps to a deeper mismatch between their ZPD and the cognitive demands of digital dialogic tasks. When story pages contained dense or rapidly delivered English narration, children were unable to integrate meaning, respond to prompts, or sustain narrative engagement. This developmental misalignment echoes Vygotsky’s (1978) claim that learning breaks down when scaffolding is not calibrated to the learner’s processing capacity.

Similarly, although young children were known to have limited attention control (Posner et al., 2014; Preece and Levy, 2020), our data showed that distraction during digital reading was technologically amplified. Children’s frequent tapping of hotspots, animations, and sound effects diverted cognitive resources away from narrative processing. This pattern is consistent with multimedia learning research showing that multimodal features imposed attentional competition that fragmented cognitive load (Kent et al., 2016) and with studies demonstrating that digital affordances disrupted parent–child talk and story comprehension (Strouse and Ganea, 2017).

These findings suggest that children’s challenges arise from ZPD misalignment, multimedia-induced cognitive overload, and the low-immersion EFL environment. This explanation highlights the unique complexities faced by young learners during digital dialogic reading in EFL families.

### 5.2 Scaffolding in dialogic Reading of digital English storybooks

Parents’ coping strategies in digital dialogic reading were shaped not only by practical needs but also by the digital EFL context. These strategies revealed that parents’ responses were adaptive, intentional, and grounded in deeper reasoning processes.

Parents’ reliance on self-scaffolding was driven by a specific psychological need to manage the linguistic anxiety, which was deeply rooted in China’s exam-oriented education legacy (Zou, 2020). Strategies such as rehearsing pronunciation or previewing vocabulary were not just preparation, but an adaptive emotional regulation process to secure their authority before facing the child (Liu, 2022; Seo, 2021; Zeng and Yung, 2025). Notably, this self-scaffolding extended to the use of emerging technologies; some parents leveraged AI chatbots to model sentences and correct pronunciation, a trend aligning with recent findings on AI-supported personalized adult learning (Alshumaimeri and Alshememry, 2023; Kang, 2023). Consistent with the adult learning perspective, parents engaged in this self-directed preparation because they were goal-oriented and needed to understand the purpose behind dialogic strategies (Knowles et al., 2005; Gordon and Ross-Gordon, 2024). Thus, self-practice served both linguistic and emotional regulatory functions to compensate for the lack of an immersive English environment.

Peer observation emerged as another important coping mechanism to distribute the cognitive load of learning new strategies. Parents who viewed others’ video recordings gained reassurance that their struggles were shared rather than personal shortcomings. This aligns with previous findings that parents in dialogic reading training often bring different expectations and anxieties than student participants (Beschorner and Hutchison, 2016). Peer modeling enabled parents to visualize abstract strategies in action and build confidence, echoing the argument that parents benefit from guided examples when learning demanding questioning strategies (Zevenbergen et al., 2018).

Bilingual scaffolding, including translation and code-switching, played a critical role in sustaining children’s comprehension and engagement. Rather than functioning as a compensatory strategy for failure, parents used translanguaging to bridge comprehension gaps and maintain narrative flow, especially when English input exceeded the child’s ZPD (Kremin et al., 2022; Nomat et al., 2024). This aligns with research showing that L1 mediation in Chinese EFL families supports engagement and reduces frustration (Hu and McKay, 2012; Carmel, 2022). By translating page-by-page or code-switching, parents functioned as strategic mediators, prioritizing interaction flow and comprehension over rigid English-only immersion.

Furthermore, parents utilized multimodal and game-based strategies to regulate attention and counteract technology-induced distraction. Recognizing the limits of young children’s attention spans (van de Ven et al., 2017), parents integrated physical activities and gamified elements, such as role-playing, into the reading process to foster intrinsic motivation (Li and Chu, 2021; Chowdhury et al., 2024). When coupled with digital affordances, such as audio narration and visual cues, these strategies helped compensate for parents’ linguistic limitations. Visual cues were particularly effective in prompting children’s responses (Eutsler and Trotter, 2020). However, parents also recognised the need to regulate the use of digital features, as excessive animations or hotspots could distract children and disrupt the dialogic flow. This is consistent with research on technoference in parent–child digital storybook interactions (Kucirkova and Flewitt, 2022; Munzer et al., 2019; Strouse and Ganea, 2017).

Finally, these coping strategies reflected parents’ attempts to navigate the cognitive load introduced by digital dialogic reading: producing English, managing dialogic prompts, and operating the device simultaneously. This is consistent with the cognitive load theory (Sweller, 1988), explaining why parents use digital tools and strategic games to distribute mental effort.

In summary, these strategies represented sophisticated, contextually grounded adaptations that illustrate how Chinese EFL parents negotiated linguistic insecurity, cognitive pressure, and digital affordances to sustain effective dialogic reading.

## 6 Conclusion

Drawing on a qualitative study of 18 Chinese parent–child dyads, this study has explored the challenges and coping strategies that emerged in the dialogic reading of digital English storybooks in an EFL learning context.

The findings revealed two major categories of challenges. Parent-related challenges were primarily characterized by the self-limitation of the communicative MKO role due to linguistic anxiety, a conflict between high cognitive load and strategy mastery, and time constraints under social pressure. Child-related challenges stemmed from a mismatch between the child’s ZPD and dialogic reading strategy demands, as well as attention competition within the digital environment.

In response, a sophisticated scaffolding system emerged. Parents engaged in self-scaffolding to function as autonomous learners, utilizing peer support and technology to develop the necessary linguistic and dialogic competence. Furthermore, dual scaffolding was established, where parents provided bilingual and contextual support while leveraging the digital storybook’s affordances to jointly facilitate children’s reading and English acquisition. This study makes significant contributions to understanding dialogic reading in digital EFL contexts. Theoretically, it extends Sociocultural Theory by complicating the traditional role of parents as MKOs. They transitioned from a position of uncertainty to becoming competent MKOs through multiple coping strategies. Furthermore, it expands the concept of Scaffolding beyond simply supporting children’s ZPD and also includes MKO’s self-scaffolding. Practically, these findings provide practical insights for implementing dialogic reading strategies in the EFL context. Parental training should be contextualized to the EFL setting and include the use of the native language and digital storybook features to empower parents. This study suggests that digital storybook Apps could be embedded with dialogic reading prompts to help guide the parent–child conversation. Furthermore, it offers confidence to parents, demonstrating that even those with limited English proficiency can use effective strategies to promote their young learners’ EFL learning.

However, this study has certain limitations. First, as a qualitative case study, the relatively small and socioeconomically homogeneous sample may limit transferability to families from different backgrounds. Second, all participants were self-identified as middle-class, and the findings may not fully represent families from other socioeconomic backgrounds. Furthermore, this study focused on parents’ perceived challenges and coping strategies rather than the frequency or quality of their specific dialogic reading strategy use, which warrants a separate, future analysis.

Future research could be expanded in several directions. Longitudinal studies could be conducted to observe how parents’ challenges and strategies evolve over time. Quantitative or mixed-methods approaches could be used to test the actual impact of different coping strategies on children’s language learning outcomes. Research could also be extended to families from different social classes or cultural backgrounds for comparative study.

## Data Availability

The datasets presented in this study can be found in online repositories. The names of the repository/repositories and accession number(s) can be found in the article/supplementary material.

## Appendix

Appendix A. Interview protocol.

1. Before this program, what was your family’s experience with reading English storybooks together?
2. What motivated you to join this digital dialogic reading program?
3. Could you share your overall experience of using digital storybooks for dialogic reading with your child over the past eight weeks?
4. What challenges, if any, did you encounter during this digital dialogic reading process?
5. When you faced these challenges, what strategies did you use to overcome them?
6. Do you think you will continue to use these dialogic reading strategies in the future, either in English or Chinese?
7. Is there anything else you would like to share about your experience that we have not talked about today?

Appendix B. Parents’ Reflective Journal.

Directions: Please fill in this form according to the titles every time after you finish dialogic reading with your child.

The child’s Name____________.

Book Title________________________;

Week ______.

**Table:**

Challenges Encountered
Coping Strategies Used
Were there any other moments you’d like to share?
